# Children's Sugar-Sweetened Beverage Consumption: Striking Parallels With Substance Use Disorder Symptoms

**DOI:** 10.3389/fped.2020.594513

**Published:** 2020-11-12

**Authors:** Allison C. Sylvetsky, Lindsey Parnarouskis, Patrick E. Merkel, Ashley N. Gearhardt

**Affiliations:** ^1^Department of Exercise and Nutrition Sciences, Milken Institute School of Public Health, The George Washington University, Washington, DC, United States; ^2^Department of Psychology, University of Michigan, Ann Arbor, MI, United States

**Keywords:** sugar, beverages, obesity, caffeine, soda

## Introduction

Sugar-sweetened beverage (SSB) intake contributes to obesity and cardiometabolic disease (1). Children's SSB consumption considerably exceeds public health recommendations (2), and efforts to reduce intake have had limited success (3). In addition to high sugar content, many SSBs also contain caffeine, and caffeinated SSBs are the predominant source of caffeine intakes among youth (4). Sugar activates central reward pathways, and similar to drugs of abuse, stimulates dopamine release (5), and meets several criteria for addiction (6). Chronic caffeine intake causes tolerance and withdrawal in children (7), which are core behavioral indicators of substance use disorders (SUDs) (6).

Compelling evidence for addictive-like responses to excess sugar intake is emerging, with accumulating support in rodent models (5). Synergistic biopsychological effects of caffeine and sugar may reinforce unfavorable beverage consumption patterns (7). SSBs are a novel stimulus from an evolutionarily standpoint, yet products containing sugar and caffeine (e.g., energy drinks) are increasingly available (8) and heavily advertised to children (7). Children have developing brains and less inhibitory control compared to adults, and thus, are particularly vulnerable to addictive substances (9). Added caffeine in already highly palatable SSBs increases their hedonic and reinforcing properties (10) and may further promote excess added sugar intakes (11).

Emerging evidence indicates that children's consumption of highly processed foods, typically high in added sugar and/or saturated fat, can lead to an addictive process reflected by core behavioral indicators of SUDs (12). These include craving, loss of control, tolerance, and withdrawal (12). Children who demonstrate more signs of addiction in their highly processed food consumption are more likely to have higher reward drive for food and higher body mass index (12). Signs of addiction have also been reported among children in response to frequent SSB consumption (13, 14). In our qualitative study (14), parents of children 8–17 years old reported that children experienced physical and affective withdrawal symptoms when caffeinated SSB intake was restricted. Similarly, Falbe et al. (13) reported that adolescents, who reported habitual SSB consumption, regardless of whether SSBs were caffeinated or caffeine-free indicated increased SSB cravings and headaches, and decreased motivation, contentment, concentration, and well-being during 72 h of SSB cessation. It is likely that other aspects of addiction (e.g., tolerance, craving, repeated unsuccessful efforts to reduce) represent important and overlooked obstacles to sustained SSB reduction. Herein, we propose that children's SSB consumption may reflect SUD symptomology and focus specifically on caffeinated SSBs, which are manufactured to contain a highly rewarding mixture of added sugar and caffeine, two ingredients that do not naturally occur in combination.

## DSM-5 Substance Use Criteria Are Highly Applicable to Children's SSB Intake

The Diagnostic and Statistical Manual of Mental Disorders, Fifth Edition (DSM-5) contains 11 criteria for SUDs (6). The DSM-5 describes a continuum of SUD severity, spanning mild (2–3 symptoms), moderate (4–5 symptoms), and severe (≥6 symptoms). Not all criteria need to be met to constitute a SUD. In our prior work in development and validation of the dimensional Yale Food Addiction Scale for Children 2.0 (dYFAS-C 2.0) (15), problem-focused symptoms (e.g., failure to fulfill obligations due to recurrent substance use) were seldom reported in children, which is consistent with other SUDs. Thus, these items were removed from the measure. In contrast, symptoms of mechanistic dysfunction (e.g., loss of control, craving, tolerance, withdrawal) were widely endorsed among children and were associated with severe eating pathology and obesity (15). Thus, over-reliance on problem-focused criteria that interfere with day-to-day functioning, which are less relevant for children, may lead to underdiagnosis of SUDs in youth even when key indicators of addictive behavior are present (15).

We examined the extent to which responses during in-depth interviews with parents (*n* = 21) (14) and focus groups with children (*n* = 37) (16) about SSB consumption (two separate cohorts, not dyads) reflected DSM-5 SUD criteria. We specifically focused on criteria pertaining to mechanistic dysfunction (Table 1). Details of the parent interviews and focus groups with children who reported daily caffeinated SSB consumption were previously published and are described elsewhere (14, 16).

**Table 1:** Parent and/or child-reported sugar-sweetened beverage consumption behaviors consistent with DSM-5 substance use disorder (SUD) criteria.

**DSM-5 criteria**	**Respondent^a^**	**Selected relevant quotations**
1. Substance often taken in larger amounts or over a longer period than was intended	Children	“After I drink one, like all of it.it makes me want another. it makes me want to pour some more [SSB] in my cup and drink that and keep going until I get tired.” “When you get some [SSB] you get like addicted to it, you just end up getting soda over and over again.”
	Parents	“They just can't stop it [drinking SSB]. For me, it is kind of like alarming.” “We literally have to like lock the drinks up cause if we don't it would all be gone the same day.”
2. Persistent desire or unsuccessful efforts to cut down or control substance use	Children	“I have [tried to cut out SSB] and I had to get it [SSB] back because it was hard for me to but I went about 4 days and then I just couldn't help it” “I've tried not drinking soda for the whole day, but I cheated.”
	Parents	“…I tried [to restrict SSB consumption] before but it wasn't working, and they [children] were like oh my goodness I need some drink.” “I tried to restrict them [SSB]…but it didn't do anything because they would bring it [SSB] back home with them.”
3. Great deal of time is spent in activities necessary to obtain or use the substance or recover from its effects	Children	“Get one [SSB] before class, and then one after class, one before class then I get one after class, then one before class again, and then before another class, I don't get one for one period, then lunch comes, and I get another one.”
	Parents	“[They drink SSB] throughout the day, yes…everything with them is sugar. Sugar is everything.”
4. Craving, or a strong desire or urge to use the substance	Children	“I just have an urge to drink it [SSB].” “…I feel like I need it [SSB]. I feel like it's something I really love and that I can't take a day without it.”
	Parents	“I can see that when they need it…like my son, he was like ‘I just need something to drink, I just need something sweet’, and I be like, ‘no’, and he says ‘but I need something sweet”’ “So it's like it's like putting candy in front of a child…Mommy has it and you're having it in front of me…I have to comment it's temptation.”
5. Substance use is continued despite knowledge of having a persistent or recurrent physical or psychological problem that is likely to have been caused or exacerbated by the substance	Children	“Soda [is] not really good for you [because of] the acid inside the soda that's like…It [SSB] can give you like kidney problems and stuff.” “If I drink too much, I get real irritated and my stomach will start hurting so I get moody”
	Parents	“The doctor said not to drink them [SSBs]; for some reason she's too activated, too much energy, and she can't sleep.” “They were complaining about their urine. They were complaining that it hurts and I'm like that's because you drunk too much soda, and it's not really like their fault because of course I bought it”
6. Need for markedly increased amounts of the substance to achieve intoxication or desired effect, markedly diminished effect with continued use of the same amount of the substance	Children	Not endorsed.
	Parents	“I think he drinks it [SSB] so much that sometimes he doesn't even get a reaction.”
7. Withdrawal syndrome or substance is taken to relieve or avoid withdrawal symptoms	Children	“I be sad because sometimes I'll still be like down and won't have no soda to bring me up.” “I'm more of a happy person [when I drink SSB], I usually make people laugh, but it's just like everything bothers me when I'm not having it [SSB].”
	Parents	“[When SSB are restricted], he'll get a little antsy, a little moody, talking at a fast pace. He will get very quiet and sometimes isolate himself.” “He gets headaches when he doesn't drink it. When he goes without drinking it for a day or two, it's something heavy and his stomach hurts, but when he starts to drink [the soda], it doesn't hurt anymore.”

a *In-depth, qualitative interviews were conducted with 21 parents of children 8–17 years of age, who per inclusion criteria, reported that their child consumed caffeinated SSBs daily (14). Focus groups were conducted with 37 children 8–14 years (n = 9 groups, each with 2–8 children), who reported daily consumption of caffeinated SSBs for inclusion (16). Interviews and focus groups were conducted by a trained moderator using a semistructured interview guide. All interviews and focus groups were audio-recorded and transcribed verbatim. Transcripts were subsequently coded independently by two coders using NVivo™*.

Behaviors consistent with 5 of the 7 DSM-5 SUD criteria pertinent to children and reflective of mechanistic dysfunction (see criteria in Table 1) were endorsed by both children and parents. One mechanistic criterion that was not widely endorsed was the need to spend a great deal of time in activities to obtain, use, or recover from the substance. This is not surprising, given that caffeinated SSBs are widely available and have a mild intoxication effect. Limited endorsement of this criterion is also consistent with findings previously reported for food and cigarettes (15). Interestingly, tolerance (#6 in Table 1) was endorsed by parents, but not by children, and may be due to tolerance being a complex concept that may not be recognized by children.

## Discussion

Consideration of SUD symptomology in future efforts to reduce children's SSB intake (and specifically caffeinated SSB intake) is warranted. For example, parallels between children's SSB consumption behaviors and well-documented patterns in SUDs further emphasize the need for beverage companies to stop marketing SSBs to youth (17). This is especially important for “non-traditional,” caffeinated SSBs such as sugar-sweetened teas, coffees, and energy drinks, sales of which have been increasing among youth (18). Incorporation of psycho-behavioral approaches used in complex and multifactorial SUDs may be useful for addressing excess SSB consumption among children. For example, children may be taught to identify situations where they experience cravings for SSB and to use self-regulation strategies to successfully reduce SSB consumption (19). Furthermore, interventions may benefit from identifying and addressing situational and contextual cues for children's SSB intake, which may result from learned associations developed over time through repeated SSB exposure (20). Addressing withdrawal symptoms or other aversive physical and affective responses when SSB intake is being reduced may be a particularly important treatment target.

Children begin consuming caffeinated SSBs at much younger ages than is typical for other addictive substances. Thus, interventions to address SSB intake require more active participation from parents than traditional SUDs. Furthermore, dietary behaviors in childhood track into adolescence and adulthood, underscoring the need to address SSB intake early in life. Reported use of SSBs to reduce negative affect is particularly concerning because intentional use of a substance to improve mood may generalize to use of other substances later in life (7). The current SUD criteria have been criticized for being context-dependent and overemphasizing problems that may arise rather than mechanisms that underpin the behavior (21). Consumption of SSBs to cope with negative emotions may serve as an important potential indicator of problematic substance use and may be particularly relevant for children, who are less likely to endorse problem-focused criteria, relative to adults (15). However, future research is required to determine the utility that adding this as a formal criteria would provide beyond the existing criteria.

Leveraging existing SUD frameworks provides a unique opportunity to enhance existing efforts to reduce children's SSB consumption. This may be especially critical for children from disadvantaged backgrounds, who are at disproportionate risk of suffering from SUDs (22) and consume the largest quantities of SSBs (2). Striking parallels between children's SSB consumption and SUD criteria emphasize the need to create and disseminate tools to identify problematic SSB consumption behaviors in high-risk children, with the goal of tailoring counseling and resources to elicit and sustain SSB behavior change.

## Author Contributions

ACS, LP, and ANG designed the project. ACS wrote the first draft of the manuscript. All authors revised the manuscript and approve of the final version submitted to Frontiers in Pediatrics.

## Conflict of Interest

The authors declare that the research was conducted in the absence of any commercial or financial relationships that could be construed as a potential conflict of interest.
